# Online and Offline Diagnosis of Motor Power Cables Based on 1D CNN and Periodic Burst Signal Injection

**DOI:** 10.3390/s21175936

**Published:** 2021-09-03

**Authors:** Heonkook Kim, Hyeyun Jeong, Hojin Lee, Sang Woo Kim

**Affiliations:** 1 Department of Electrical Engineering, Pohang University of Science and Technology, 77 Cheongam-Ro, Nam-Gu, Pohang 37673, Korea; kimhk85@postech.ac.kr (H.K.); jhy90@postech.edu (H.J.); suvvus@postech.edu (H.L.); 2 Hyundai Robotics Co., Ltd., 50, Techno Sunhwan-ro 3-gil, Yuga, Dalseong-gun, Daegu 43022, Korea

**Keywords:** cable fault, 1D CNN, soft faults, industrial robots, online detection

## Abstract

We introduce a new approach for online and offline soft fault diagnosis in motor power cables, utilizing periodic burst injection and nonintrusive capacitive coupling. We focus on diagnosing soft faults because local cable modifications or soft faults that occur without any indication while the cable is still operational can eventually develop into hard faults; furthermore, advance diagnosis of soft faults is more beneficial than the later diagnosis of hard faults, with respect to preventing catastrophic production stoppages. Both online and offline diagnoses with on-site diagnostic ability are needed because the equipment in the automated lines operates for 24 h per day, except during scheduled maintenance. A 1D CNN model was utilized to learn high-level features. The advantages of the proposed method are that (1) it is suitable for wiring harness cables in automated factories, where the installed cables are extremely short; (2) it can be simply and identically applied for both online and offline diagnoses and to a variety of cable types; and (3) the diagnosis model can be directly established from the raw signal, without manual feature extraction and prior domain knowledge. Experiments conducted with various fault scenarios demonstrate that this method can be applied to practical cable faults.

## 1. Introduction

With the growth of fully automated production lines in modern manufacturing, the early detection of faults in the motor power cable of automation machinery has become a demanding requirement to reduce unscheduled maintenance. A single hard fault in the motor cable could result in unplanned production line downtime because the machines need to be taken offline to ensure safety during inspection. Depending on the industry, this catastrophic production line stoppage could result in a huge loss of productivity. For this reason, the integrity of the motor cables in manufacturing automation, such as automotive factory floors, must be ensured for safe operation. Therefore, online diagnostics of cables are crucial, because unscheduled maintenance occurs when the machine is working; thus, the suspicious cable cannot be isolated from production lines but exhibits intermittent fault symptoms. Offline diagnostics are still required for regular maintenance when the machine can be offline for preventive maintenance.

In factory floors, power cables with connectors are installed close to vibrating motors and welding machines; these have high current consumption and produce heat. The harsh industrial environment around cables can cause aging degradation of the cable [1], resulting in cable faults, i.e., soft and hard faults. Local modifications to the cable or soft faults due to the stressful environment can, while the cable is still working and without any indication, be transformed into hard faults by subsequent partial component damage, e.g., to the cable conductors, coatings, and shield [2]; this makes it difficult to observe ongoing aging damage to the cable.

Cable fault diagnostic methods have been broadly studied and applied in many fields, such as automotive engineering [3], power plants [4], ship buildings [5], and aircraft [6]. Nowadays, cable condition assessment for factory automation has become significant to increase productivity by decreasing downtime. There are several requirements for the methods to be applicable in automated factory fields, as follows:

(1) Early detection of soft faults before they transform into hard faults.
(2) Applicable to very short cable.
(3) Both online and offline diagnostic ability with an identical scheme for unscheduled and scheduled maintenance.
(4) No manual feature extraction and no prior knowledge.
(5) Nonintrusive and nondestructive diagnostic techniques.
(6) Runnable with limited computing resources (for machine controllers).

We conclude that there are no perfect traditional or recently studied methods for cable diagnosis in the field of automated factories.

Traditional cable condition monitoring methods can be largely categorized into mechanical (elongation at break), chemical (oxidation induction time), and electrical techniques (partial discharge) [7]. These techniques are limited in on-site diagnostics because only some cable parts are accessible on the factory floor. Moreover, online diagnosis of on-voltage cables is not possible using these methods because the machine needs to be turned off for inspection and online partial discharge methods are too sensitive to noise and time [1].

Over the last decade, reflectometry-based methods—such as time domain reflectometry (TDR), frequency domain reflectometry (FDR), and (joint) time–frequency domain reflectometry (TFDR)—have been introduced as effective methods for cable diagnostics [3,4,5,6,7,8,9,10,11,12,13,14,15]. TDR uses an impulse signal as an incident signal and analyzes the reflected signal to diagnose and locate faults in the time domain. In contrast, FDR analyzes the reflected signal in the frequency domain using a set of sinusoidal signals as an incident signal. Moreover, TFDR diagnoses faults based on the time–frequency domain using a Gaussian-enveloped linear chirp signal as the incident signal. Among these advanced reflectometry-based methods, TFDR demonstrates the robust capability of fault diagnosis on various cables because TFDR has characteristics in both time and frequency domains. Although these techniques tend to effectively diagnose hard faults, owing to their huge reflection, they cannot always diagnose small anomalies, such as soft faults. Owing to the physical limitations intrinsic to how soft faults respond to incident signals, the extension of TDR from hard faults to soft faults needs to be further considered before applying them to practical problems [2]. Moreover, to accurately diagnose soft faults using TDR, FDR, and spread spectrum TDR (SSTDR) that utilizes the sinusoidal PN sequence to diagnose live wires, Ref. [16] suggested that the impedance variation in the environment of the wiring system must be smaller than that due to the soft fault. Primarily, these reflectometry-based methods need to address proper compensation of the reflected signal, because the propagated signal experiences time dispersion and amplitude attenuation.

In addition, the accuracy of reflectometry-based methods can be degraded by blind spots for excessively short fault distances when the incident and reflected signals overlap [17]. Blind spots can be a fundamental issue when applying reflectometry-based diagnostics. To the best of the authors’ knowledge, these methods did not sufficiently focus on extremely short cables; thus, they cannot guarantee high diagnosis accuracy for cables used in automated factories. Unlike the power cables in power plants, where the power transmission line length is in the range of kilometers, those in automotive factories and factory automation machines are extremely short and have fixed lengths according to the factory layout and manufacturers’ specifications.

Online detection of cable faults has been studied for fault detection in aircraft during flight [6]. This method demonstrates that the proposed spread-spectrum time-domain reflectometry (SSTDR) is suitable for online detection and performs better than spectrum time-domain reflectometry (STDR) because of the wider spectrum and sharper correlation peak. However, this method is not suitable for soft fault detection and offline detection. In [18], the authors proposed a cable diagnosis based on online monitoring of the metal sheath current to identify cable sheath faults in power distribution networks. This approach allows an online fault diagnosis of the metal sheath of the HV cable. However, this method requires link boxes for cross-bonded metal sheath connections for each section of the HV cable. In addition, this method cannot diagnose the soft faults in the conductor and cannot conduct an offline diagnosis.

Some studies have demonstrated that the baseline method using the resultant signal of the faulty cable to compare with the signal from the normal cable could detect and locate soft faults [16]. This approach is thought to be suitable and precise for soft faults; however, a perfect baseline signal is difficult to obtain, because the resultant signal—from even a healthy cable—can vary according to the installation conditions, machine vibration, and cable movement. Moreover, this method requires manual feature extraction and prior knowledge during the analysis of the input and resultant signals. In addition, online detection of cable faults is not possible for this method because the baseline signal changes under the influence of the motor control signal and incident signal.

In this paper, a new data-driven approach to satisfy all required demands based on the periodic burst injection (PBI) method and 1D convolutional neural network (1D CNN) is proposed for the early detection of soft faults. The 1D CNN has a simple structure, with a small number of learnable parameters and sensitivity to one-dimensional time series. In addition, we utilize the resultant signal rather than the reflected signal to eliminate the blind spot issue and diagnose very short cables. First, an incident signal is injected into the cable using nonintrusive capacitive coupling, and the resultant voltage signal is then collected from the other end of the cable to extract features from soft faults. The sequence of distinct pulses is utilized as the periodic incident signal to extract features from the faulty cable, even when the machine is working.

The rest of this paper is organized as follows. The theoretical background of the diagnosis model is introduced in Section 2. Section 3 and Section 4 demonstrate the algorithm, using three cable settings. Finally, Section 5 presents the achievements and conclusions of this study.

## 2. Theory of Fault Detection Method

### 2.1. Cable Soft Fault and Degrees of Severity

Advance diagnosis of soft faults is more beneficial than the later diagnosis of hard faults, because soft faults have a high probability of becoming hard faults. In addition, these days hard faults can be detected from error codes generated by automated machines, such as industrial robots, which are designed to produce errors when hard faults are detected, alerting the maintenance staff and entering a safe state until the fault is cleared [19]. For example, if a hard fault, such as an open circuit, occurs on the servomotor power cable that controls the third servomotor of an industrial robot, the robot will generate an error code that describes the cause of failure, for example, power loss in the third servomotor. The maintenance staff can then analyze the cause of failure—e.g., an open circuit in the third power cable—to repair the robotic system. This implies that hard faults are easy to diagnose if they have already occurred; thus, we need to focus on diagnosing soft faults in the case of automated machines.

Fault localization is a good way to analyze the cause and improve the cable installation process. However, to immediately restart the production lines, the faulty cables tend to be replaced first and analyzed later; this is because wiring harness cables in the field are very short and easy to disconnect, owing to heavy-duty connectors, which allow easy locking and unlocking at both ends of the cable. Therefore, diagnosing soft faults and taking proper measures at an early stage is the best strategy for reducing downtime.

To simulate soft fault damage with various degrees of severity, we gradually increased the wire characteristic impedance by reducing the cross-sectional area of the target wire while fixing the damage length to 40 mm. We denote the cross-sectional area of the normal wire as So, and the remaining cross-sectional area, which corresponds to the damaged wire, as Sf. Sf and So are related by Sf = α So, where α is the ratio of the damaged area with 0 < α ≤ 1. For the experiment, we varied α from 1.00 to 0.066, while maintaining a fixed soft damage length to regulate the damage level. We demonstrate that the early stage of soft fault, i.e., the small α, is detectable by the proposed method in the experiment section. The hard faults cases, which consist of open circuit (α = 0) and short circuit (α > 1), are not considered in this paper.

**Figure 1:**
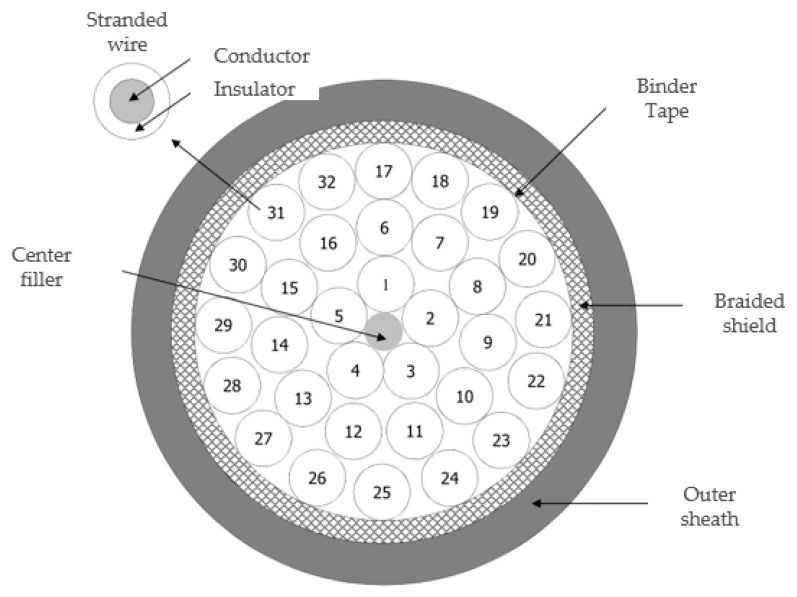
Cross-sectional view of motor power cable (COVV-SB 32C × 1.5SQ).

**Figure 2:**
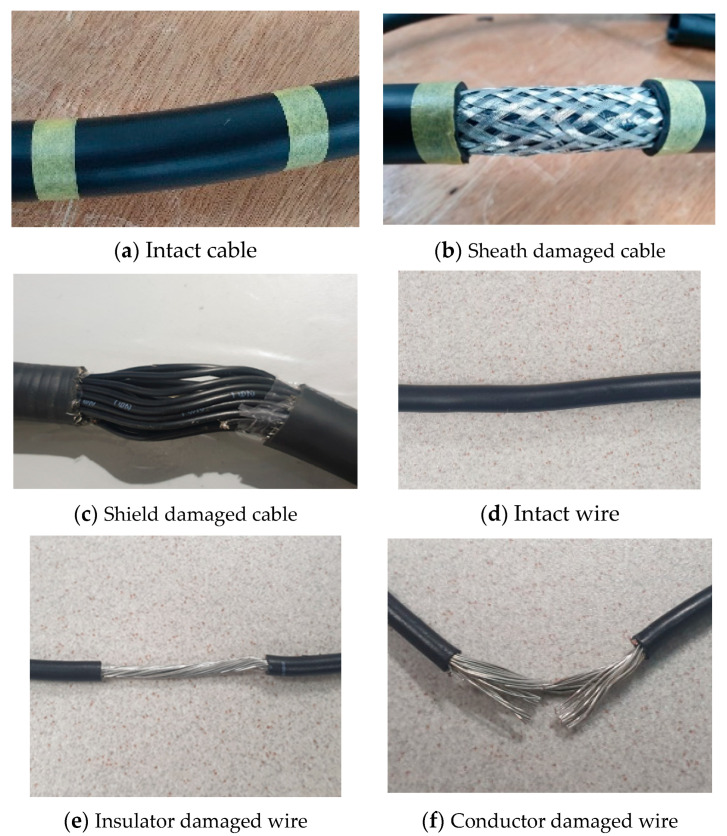
(**a**) A normal cable with intact sheath. (**b**) A faulty cable without sheath (the braided shield is visible). (**c**) A faulty cable without shield (the wires are visible). (**d**) A normal wire inside the cable. (**e**) A faulty wire without an insulator (the conductor is visible). (**f**) A faulty wire with damaged conductor (some strands are cut).

The target cable in this study was a COVV-SB 32C × 1.5SQ cable from the LS cable. A cross-sectional view of the cable is presented in Figure 1. It has 32 stranded wires, which are composed of small wires wrapped together to form a larger conductor to resist metal fatigue. Each stranded wire consists of 30 strands of 0.25 mm small-diameter wire, with a total cross-sectional area of 1.5 mm^2^. The soft fault cases in this study comprised shield and conductor damage. Shield damage refers to damage to the sheath and braided shield, as shown in Figure 2b,c. For the shield damage case, the sheath and braided shield were removed cleanly in this study. Conductor damage refers to damage to the insulator and conductor, as shown in Figure 2e,f. The cross-sectional area of the stranded wire can be reduced as α decreases by removing a fixed damage of length w of the conductor in each strand.

### 2.2. Periodic Burst Signal

The burst signal is a sequence of a limited number of distinct pulses or an oscillation of limited duration [20]. In the proposed method, this signal is used as the incident signal to extract as many features as possible. The repeated incident signal was injected into the on-voltage motor power cable at a specific frequency. However, the performance of the robot manipulator will not be degraded because the robotic system is immune to this kind of fast transient disruptive signal, as verified during the prototype stage of the manipulator in accordance with the international EMC standard [21,22]. Immunity tests are mandated by the international EMC standard. Therefore, industrial robots installed in the manufacturing field can be regarded as having passed these tests and possess immunity. Protective measures to protect control circuits from fast transient disruptive signals are incorporated into industrial robots. Therefore, the burst signal with a specific condition will not cause any functional degradation of the target system, regardless of whether the machine is online or offline. By analyzing the extracted features, it is possible to build an anomaly detection algorithm that detects a variety of soft faults.

The characteristics of the burst pulses were defined in the international EMC standard. A mathematical description is presented in [23,24]. The time dependence of the voltage is expressed as:

(1) $$v_{EFT}=k_{v}\left[\frac{V_{1}}{k_{EFT}}\cdot \frac{{\left(\left.\frac{t}{\tau_{1}}\right)\right.}^{n_{EFT}}}{1+{\left(\left.\frac{t}{\tau_{1}}\right)\right.}^{n_{EFT}\;}}\cdot e^{\frac{-t}{\tau_{2}}}\right]$$

where *k_v_* is the peak voltage value under no-load condition and *k_EFT_* is:

(2) $$k_{EFT}=e^{-\frac{\tau_{1}}{\tau_{2}}\cdot \left(\frac{n_{EFT}\cdot \tau_{2}}{\tau_{1}}\right)}{}^{\frac{1}{n_{EFT}}}$$

where *V*_1_ = 0.92, *τ*_1_ = 3.5 ns, τ_2_ = 51 ns, and *n_EFT_* = 1.8, according to the EMC standard.

**Figure 3:**
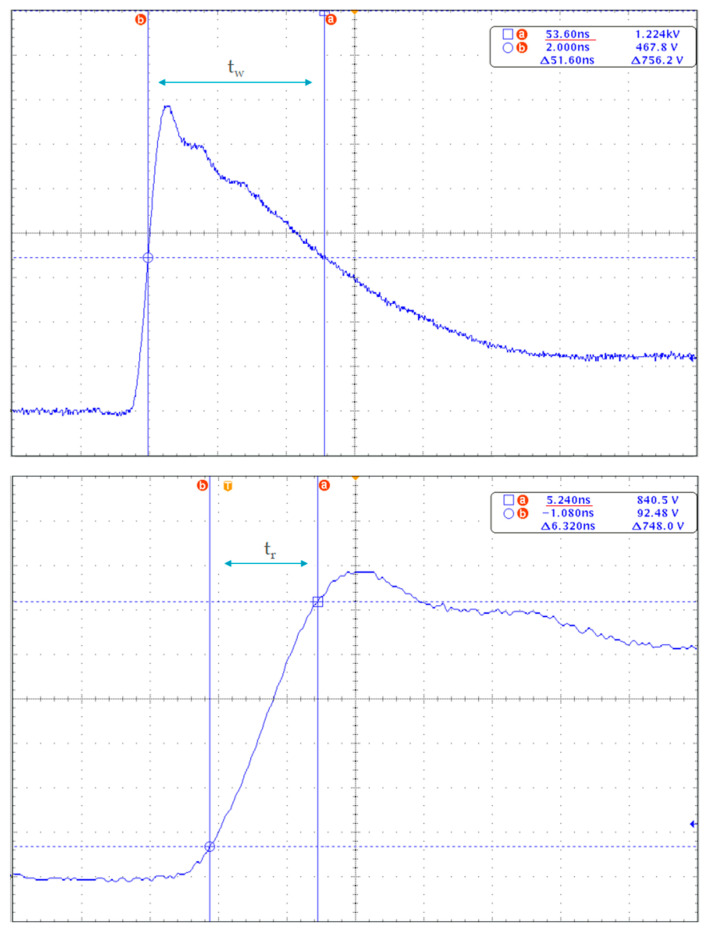
The ideal waveform of a single pulse into a 50 Ω load with nominal parameters t_r_ = 5 ns and t_w_ = 50 ns.

According to the EMC standard, the burst signal should have a rising time of 5 ns and a width of 50 ns, with some tolerance, as shown in Figure 3. These relatively quick nanosecond-scale pulses should be repeated for durations of 15 ms or 0.75 ms (Td), and frequencies of 5 kHz or 100 kHz (Fs), for a period of 300 ms (Tr).

**Figure 4:**
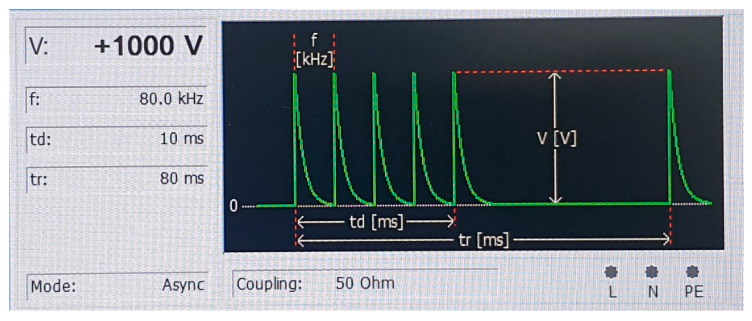
Representation of the incident periodic burst signal.

Figure 4 shows the sequence of burst signals for the use of the incident signal. For feature extraction, we designed a burst signal with Fs = 80 kHz, Td = 10 ms, and Tr = 80 ms, which were empirically determined to allow the extraction of many features from the fault response. The peak amplitude was set to be 1 kV.

### 2.3. Nonintrusive Capacitive Coupling and Periodic Burst Injection (PBI) Method

The intensity of the reflected signal is strongly affected by the bandwidth of the incident signals and the length between the incident point and soft fault. The resultant signal is similarly affected by passing through the faulty cable. A larger reflection of the incident burst signal reduces the intensity of the resultant signal. In this study, the bandwidth of the incident signal was determined using the periodic burst signal design process described in Section 2.2.

A capacitive coupling clamp (CCC) can be utilized to inject nanosecond burst pulses into a live (online) or dead (offline) target cable [25]. In the conventional method, the incident signal is typically injected directly into the cable. Directly injecting the incident signal can be unsafe and requires a protective circuit to protect the machine and maintenance people, because the live target cable, which delivers motor power to the robot manipulator, carries a lethal amount of current. Moreover, production lines cannot operate around the unplanned downtime by turning off the machine so that the wiring harness cable can be disconnected from the machines to directly inject the burst signal. Therefore, a suspicious cable can be diagnosed without interrupting plant operation via a capacitive coupler.

**Figure 5:**
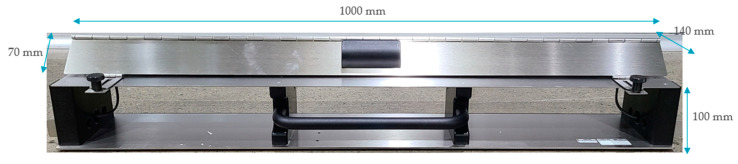
Architecture of the capacitive coupling clamp (CCC).

The coupling efficiency of a CCC is determined by the coupling capacitance between the target cable and clamp. CCC conforms to the specifications of international standards, as shown in Figure 5. This guarantees that each diagnosis can be conducted under identical conditions. The target cable was placed inside the clamp and the coupling plates were closed for capacitive coupling. The burst generator was then connected to either side of the coupling clamp.

**Figure 6:**
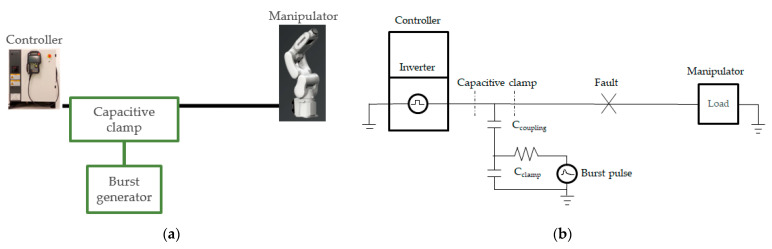
(**a**) Nonintrusive periodic burst signal injection method. (**b**) Equivalent circuit.

Figure 6 shows a diagram of the nonintrusive periodic burst injection and its simplified equivalent coupling circuit. Here, C_clamp_ is the capacitance of the clamp and C_coupling_ is the coupling capacitance between the clamp and target cable. The circuit can be simplified, as shown in Figure 6b, because the dominance of the capacitive coupling between the clamp and cable in the coupling mechanism allows the clamp inductance to be ignored [24].

**Figure 7:**
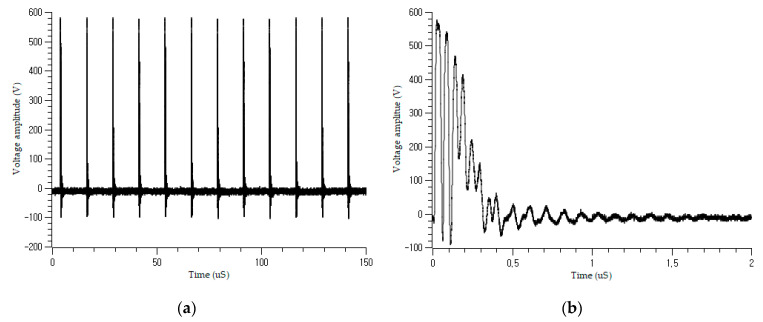
Offline burst signal injection on the cable: (**a**) coupled periodic burst signal; (**b**) a single pulse from (**a**).

**Figure 8:**
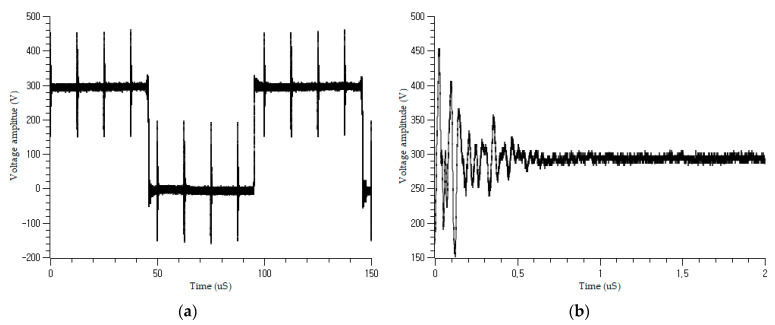
Online burst signal injection on the cable: (**a**) coupled periodic burst signal; (**b**) a single pulse from (**a**).

The coupled incident signals are shown in Figure 7. The actual signal differs from the ideal signal in Section 2.2, owing to the effect of the existing shield and the variation in the impedance with cable condition. The large pulse contains numerous small pulses that decay over time, as shown in Figure 7b. Figure 8 shows the coupled signal in the on-voltage motor power cable when the robot is working. There are fluctuating PWM power signals in the figure, which are generated by the controller inverter to control the servomotors of the robot manipulator. The burst signal can be autonomously isolated in the input layer and delivered to the learning network. The main frequency of the motor control signal was 9 kHz, whereas the frequency of the pulses was 80 kHz, with a duration of 10 ms and a period of 80 ms.

## 3. Proposed Soft Fault Diagnosis Method Using PBI and 1D CNN

**Figure 9:**
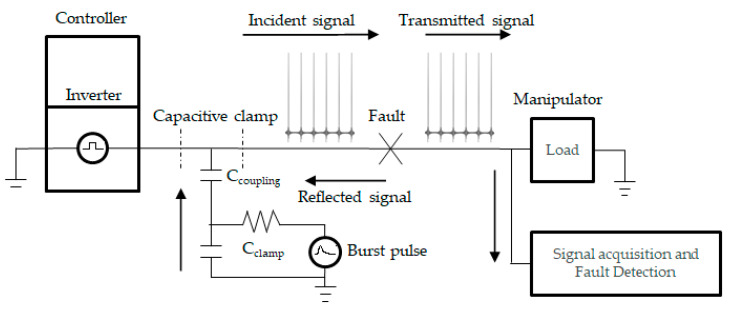
PBI cable fault diagnosis.

**Figure 10:**
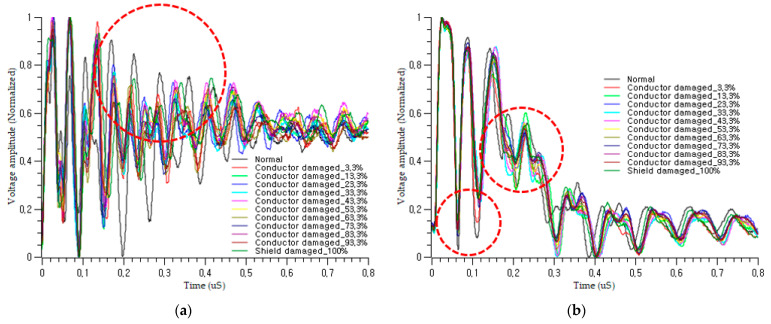
Transmitted signals through the faulty cable with various severity degrees of fault: (**a**) pulses detected online; (**b**) pulses detected offline.

Figure 9 shows the architecture of the cable fault diagnosis model using nonintrusive PBI. The signal is injected into the cable using a CCC. According to the law of reflection, when the incident signal is transmitted to the fault point, some of the signal is reflected, depending on the reflection coefficient, and the remainder is transmitted to the detection device at the other end of the cable. The detection device then extracts features and classifies the state of the cable condition using a 1D CNN. In this study, the detectable soft faults are the damage to the shield and conductors, with varying degrees of severity. Damage to the cable changes the transmission parameters and, thus, changes the shapes of the reflected and transmitted signals, as shown in Figure 10. The pulse shapes in the online and offline cases differed according to the severity of the fault. The voltage amplitude and traveling time of the bust signal changes as the degrees of severity change.

The need for manual feature extraction and prior knowledge when analyzing the resultant signal makes it hard to extract the deep characteristics of signals from various types of motor cables in a factory. Moreover, the computing power of machine controllers, such as programable logic controllers (PLCs) from automated factories, is very limited; even if they have a CPU and memory, they are only enough for the normal operation of machines. Therefore, recent works that use complicated algorithms running on high-performance computers cannot be applied in this field.

The use of a simple 1D CNN for soft fault diagnosis can overcome this problem. A CNN model utilizes multiple convolutional layers to extract higher-level features from the raw input signal. Compared with other CNN-based models, a 1D CNN model has a simpler structure and guarantees high classification accuracy using a restricted training dataset with sensitivity to one-dimensional time series signals [26,27]. In addition, the kernels of a 1D CNN can be properly set using only a small number of back-propagation epochs, thereby making the 1D CNN fast enough to apply for various types of motor power cables from automated machines, which have a varied capacity of motors, and motor power cables with proper conductor thickness. For example, cables with thicker conductor are needed for the motors with greater capacity. Therefore, 1D CNN is adopted as a classifier for the proposed method, because it has a relatively small number of learnable parameters so that the time for the learning process can be shortened and the machine controller with limited computing resources can conduct this algorithm on-site.

**Figure 11:**
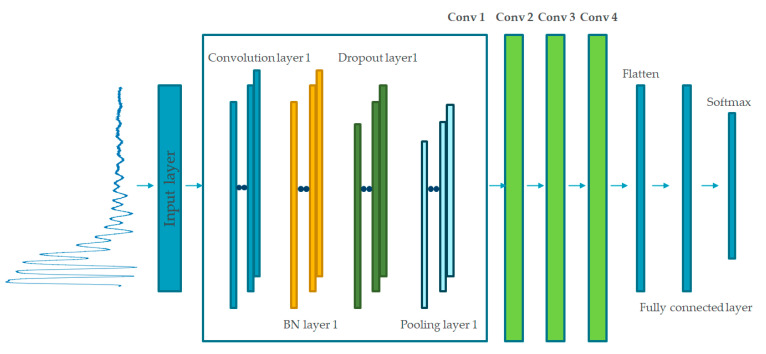
Construction of the proposed 1D CNN model.

The proposed 1D CNN consists of convolutional layers, batch normalization layers, dropout layers, max-pooling layers, fully connected (FC) layers, and a softmax classifier, as shown in Figure 11. The architecture allows one-dimensional feature extraction without handcrafted manipulation. A batch normalization layer was followed by a dropout layer.

The batch normalization layer allows the proposed model to achieve higher validation and test accuracy on datasets with stable gradient propagation, whereas the dropout layer was adopted to avoid overfitting. A softmax classifier is adopted after the fully connected layers to classify the data into two classes, i.e., normal and fault.

The voltage amplitude of each transmitted periodic burst signal was sampled as 1D time-series data using a data acquisition device. Then, several pulses from this raw signal were captured, normalized, and loaded into the next convolution blocks. The dimensions of the captured signal were 3000 × 1, at a sampling rate of 5 GHz, as per the proposed architecture. To generalize the diagnosis model and reduce the effect of different parameter values, such as the locations of the clamp/fault and the length of the cable, an affine transformation is applied to the training dataset as a data augmentation method. Affine transformations preserve collinearity and distance ratios [28], and include geometric contraction, expansion, dilation, reflection, rotation, shear, similarity transformations, spiral similarities, and translation. Here, a randomly changing scale was adopted for affine transformation.

**Figure 12:**
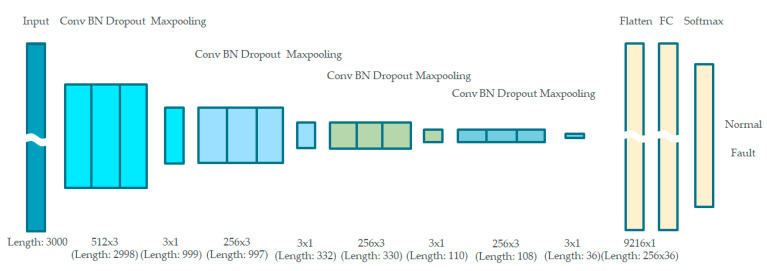
Detailed configuration of 1D CNN model.

The parameters of each layer must be properly set according to the type of cable and classification task. The detailed architecture of the proposed online and offline 1D CNN model is shown in Figure 12. The four convolutional blocks with different parameters are connected in series to learn the hierarchical features of the fault response. The first convolution block has 512 kernels, and the second, third, and final convolution blocks each have 256 kernels. The convolution kernel sizes were fixed at three for all blocks. This hierarchical architecture reduces the number of learnable parameters in each convolutional block and enables the diagnosis model to learn low- to high-level features. ReLU was used as the activation function of each convolution block, and the batch size was fixed at 20.

## 4. Experimental Setup

**Figure 13:**
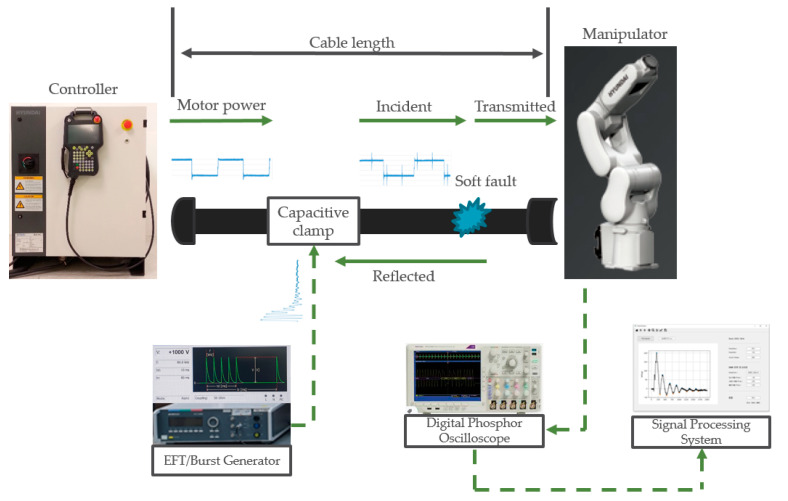
Schematic diagram of the fault diagnosis system.

Figure 13 shows the proposed fault diagnosis system, which comprises an incident signal-generating part, a signal measurement part, and a signal-processing part. The burst generator created the designed incident signal described in Section 2.2. This signal is coupled to the target cable using a nonintrusive CCC. The transmitted signal was measured using a digital phosphor oscilloscope (DPO) at the end of the cable. Then, the signal-processing system collects the measured signal and performs 1D CNN classification based on the time sequence raw signal.

An Emtest UCS 500N was used as a burst generator to generate the designed incident signal, and a Tektronix DPO 4104 B phosphor oscilloscope was used to acquire the transmitted signal at a sampling rate of 5 GHz. An Emtest HFK was used as the CCC to couple the burst signal to the cable. A Hyundai Hi5a-T10 industrial robot controller was used to control the Hyundai HH7 manipulator with a payload of 7 kg. To collect the output signal in this study, we used the test point located in the junction box of the manipulator. Using this test point ensures accurate collection of the fault response under normal and faulty conditions. For offline fault detection, the burst signal was injected into the target cable while the controller was turned off and the manipulator remained stationary. For online fault detection, the controller was turned on so that the inverter inside the controller generated power and delivered it to the robot manipulator through the wiring harness cable. A burst signal was then injected into the target cable. The target cable consists of 32 wires, and the burst was injected into all the wires.

**Figure 14:**
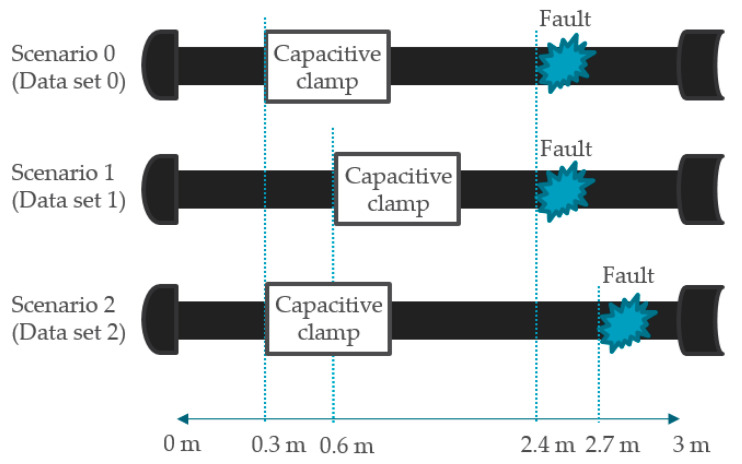
Three scenarios of simulated conditions.

To verify the feasibility of the proposed algorithm for offline and online cable fault diagnosis, we conducted experiments corresponding to the three scenarios, as shown in Figure 14, under different locations of the clamp and fault. We also prepared an intentionally damaged cable with one level of normal condition and 11 levels of damage severity on the third and fourth wires of the cable. The conductors in these target wires were intentionally damaged by cutting out the strands, reducing the cross-sectional area of the conductor. The severity of each damage level is listed in Table 1. There were, therefore, a total of 72 cases corresponding to 12 levels of cable condition (one normal and 11 faulty) × 3 settings × 2 wires. For each case, at least dozens of pulses were transmitted, because the burst generator sent consecutive periodic pulses with an 80 ms period for 2 min for each diagnosis. This allowed sufficient features to be obtained from the pulse train on each diagnosis, facilitating the learning process by providing sufficient raw data to the input layer.

The length of the prepared cable was 3 m and the cable ends were connected to a heavy-duty connector. In the first scenario, for dataset 0, we placed 11 degrees of soft fault at 2.4 m along the cable and the CCC at 0.3 m along the cable. In the second scenario, for dataset 1, we placed 11 degrees of soft fault at 2.4 m along the cable and the CCC at 0.6 m along the cable. In the last scenario, for dataset 2, we placed 11 degrees of soft fault at 2.7 m along the cable and the CCC at 0.3 m along the cable. The positions of the capacitive clamp in the first scenario differ from those in the second scenario so that the effects of different clamp positions with the same fault position could be learned. In addition, the positions of the fault position in the first scenario differ from those in the third scenario so that the effects of different fault positions with the same clamp position could be learned.

The experimental data obtained from these scenarios are summarized in Table 2. Dataset 0 was used as the training and validation sets for this study. Datasets 1 and 2 were used as test sets.

## 5. Results and Discussion

### 5.1. Offline Detection Results

The robot controller was turned off to conduct an offline fault diagnosis. The controller, manipulator, and wiring harness cables between them were installed under the same conditions for online fault diagnosis. The transmitted pulses were collected by the DPO, and each pulse was isolated and delivered to the learning process.

Dataset 0 in Table 2a was used as the training and validation sets for the 1D CNN. Datasets 1 and 2, which had different fault locations and clamp locations from dataset 0, were used as the testing sets. The results obtained from the 1D CNN classifier are presented in Table 3.

One normal mode and 11 gradually increasingly damaged conductor modes, including the damaged shield at the outer part of the cable, were classified correctly. The average classification accuracy of the 1D CNN was 98.97% for dataset 1 and 98.76% for dataset 2. Each dataset had a different setting for CCC and fault location. Random affine transformation, with scales varying from 0.95 to 0.99, was applied for data augmentation. The data augmentation method reduced the effect of different settings and improved the accuracy through generalization. Therefore, even though learning dataset 0 had a different setting, the 1D CNN algorithm could effectively classify the abnormalities in datasets 1 and 2.

From the experimental results, it is clear that the proposed cable fault diagnosis using PBI and capacitive coupling detected the abnormalities correctly. Even for the early stage of soft fault cases, which include the damage of the shield and the damage of a small portion (α = 3.3%) of the conductor, the proposed method was diagnosed well. Therefore, we could first detect the shield ruined by the harsh environment, and the beginning of the conductor damage after the shield was totally damaged, because the reduced cross-sectional area of the conductor is detectable too. The minimum decrease in the cross-sectional area of a conductor in the experiment was 3.3% and it was detected well. In addition, the normal condition was well diagnosed. This verifies the feasibility and effectiveness of the proposed method.

### 5.2. Online Detection Results

The robot controller was turned on to move the manipulator as programed for online fault diagnosis. The controller, manipulator, and harness cable running between them were installed under the same conditions as in offline fault diagnosis, except that the six servomotors of the manipulator were powered on and the harness cable carried a voltage.

The incident burst signal was the same as that in the offline experiment. In addition, there was a PWM squared base signal of approximately 400 V amplitude due to the control signal delivered from the inverter of the robot controller to the manipulator through the wiring harness cable. The controlled pulse width changed according to the command from the CPU, but the burst was still detectable. The pulse capture unit in the input layer of the algorithm can capture the pulse signals from the squared signal, as shown in Figure 8.

Dataset 0 from Table 2b was used as the training and validation sets for the 1D CNN, and datasets 1 and 2, with different fault positions and clamp positions, were used as the testing sets. The results obtained from the 1D CNN classifier are presented in Table 4.

The fault classification results indicate that the proposed 1D CNN method can be applied to automated production lines for fault diagnosis, even when the robot is operating, that is, when the power generated by the inverter of the robot controller is being delivered to the robot manipulator. Random affine transformation, with random scales varying from 0.95 to 1.00, was applied for data augmentation. The average classification accuracy of the proposed algorithm was 98.00% for dataset 1 and 89.00% for dataset 2. The detection accuracy of the normal condition was 95–97%, which is sufficiently high for use in actual applications.

The overall accuracy of dataset 1 was higher than that of dataset 2, because the accuracies for damage severity in dataset 2—from 53.3 to 63.3%—were relatively lower than those of the other damage cases. In addition, the damage accuracies for the other damage severities—from normal to 43.3% and from 73.3 to 93.3%—were sufficiently high to detect an ongoing soft fault in the target cable, even in the presence of a low-accuracy region, because a cable tends to become gradually damaged over time and external stresses are accumulated in the cable until the damage becomes severe. Therefore, the algorithm can detect ongoing soft fault damage in the fault lifecycle as the soft fault develops from the normal condition, through the early stage (with minimum damage), to the highest damage severity. In this experiment, the highest damage severity of conductor was the removal of 93.3% of the cross-sectional area of the target wire.

### 5.3. Comparison with Other Methods

To evaluate the abilities of the proposed fault diagnosis method, the proposed method was compared with other fault diagnosis methods, as shown in Table 5. The mechanical techniques, such as the elongation at break (EAB), are considered classical cable diagnosis methods, but they are destructive, and both specialized training and experience is required to operate the polymer tensile testing machine. On the other hand, the chemical techniques include the oxidation induction time (OIT), which is a measure of the time at which rapid oxidation of a test material occurs in a flowing oxygen environment. The measured time can be correlated to the remaining life of the target cable by estimating the level of antioxidant, but OIT test needs a sample of the target cable to diagnose its condition, because it needs to be performed in a laboratory setting only. This can be destructive and undesirable. The electrical techniques, such as partial discharge (PD) and dielectric loss (tan δ), are representative diagnosis methods for field testing. However, the PD test is susceptible to noise and requires disconnecting the cable terminations from automated machines. Interpretation of the test results requires an extremely high skill level. Besides, tan δ measurement can be unreliable on unshielded cables because of irregular ground path. The reflectometry-based methods (TDR, FDR, TFDR, SSTDR) need to address proper compensation of the reflected signal due to time dispersion and amplitude attenuation. Moreover, the accuracy of these methods can be degraded by blind spots for excessively short cables when the incident and reflected signals overlap.

The most significant difference between the previous methods and the proposed method are that (1) it needs no prior domain knowledge; (2) it can diagnose both offline and online cables; and (3) it can be applied to very short cables. The proposed method demonstrates that soft fault diagnosis can be achieved under various operating conditions, such as automated machines having different types of cables in the automotive factories.

## 6. Conclusions

Motor power cables play an important role in automated manufacturing facilities. Cable faults can result in a costly reduction in productivity and customer trust and an increase in workplace injuries, because safety devices and industrial equipment are operated through cables. A new and effective approach is needed to diagnose extremely short cables and conduct online and offline diagnoses with the same architecture. For applicability to the factory floor, diagnosis methods with few parameters and no manual manipulation are required, in contrast to other methods, which require prior knowledge of the prerequisite parameters and raw data preprocessing involving special skills. A new method of satisfying these requirements, based on a data-driven approach using a compact 1D CNN model and nonintrusive PBI, is proposed in this paper.

A Hyundai HH7 robot manipulator and controller were used for the experiment in this study. A burst generator produced an incident signal, which was coupled to the harness cable between the robot manipulator and the controller by a nonintrusive CCC. The transmitted signal was measured using a DPO at the end of the cable. Then, the signal processing system collected the resultant signal and performed a 1D CNN classification. The average classification accuracy on datasets 1 and 2 was sufficient for practical use in diagnosing actual cable issues. For example, offline diagnosis can be used when the robot is turned off for scheduled maintenance and online diagnosis can be used while the robot is operating; however, emergency diagnosis is needed because of intermittent faulty symptoms in the live cables.

In future work, the real-time fault diagnosis of a U-motion moving cable is a suitable but challenging problem. The main drawback of the currently proposed method is that a large number of accurate datasets are required to ensure classification accuracy. To overcome this problem, the applied data augmentation method needs to be developed and expanded. In the near future, we will focus on data augmentation methods to improve diagnostic accuracy.

## Figures and Tables

**Table 1:** Degrees of damage severity.

# of Strands Cut (Out of 30)	Degree of Conductor Damage	Degree of Shield Damage
1	3.3%	-
4	13.3%	-
7	23.3%	-
10	33.3%	-
13	43.3%	-
16	53.3%	-
19	63.3%	-
22	73.3%	-
25	83.3%	-
28	93.3%	-
Remove all	-	100%

**Table 2:** The number of collected datasets for Data 0, Data 1, and Data 2. Each dataset is measured according to the 3 scenarios from Figure 14: (**a**) offline data set; (**b**) online data set.

Dataset	Damage Severity Degrees											
	Normal	Conductor (1- α in Percentage)										Shield
	0%	3.3%	13.3%	23.3%	33.3%	43.3%	53.3%	63.3%	73.3%	83.3%	93.3%	100%
# of Data 0	48	48	48	48	48	48	48	48	48	48	48	48
# of Data 1	608	576	576	576	576	576	288	576	576	576	576	576
# of Data 2	576	642	642	642	642	642	642	642	642	642	642	576
(**a**)												
**Dataset**	**Damage Severity Degrees**											
	**Normal**	**Conductor (1- α in Percentage)**										**Shield**
	**0%**	**3.3%**	**13.3%**	**23.3%**	**33.3%**	**43.3%**	**53.3%**	**63.3%**	**73.3%**	**83.3%**	**93.3%**	**100%**
# of Data 0	22	24	24	24	24	24	20	24	24	20	20	24
# of Data 1	252	287	288	288	288	288	320	288	252	288	288	288
# of Data 2	288	319	279	287	318	318	318	318	300	318	318	288
(**b**)												

**Table 3:** Offline fault detection accuracies of soft faults in the cable.

Dataset	Damage Severity												Overall
	Normal	Conductor (1- α in Percentage)										Shield	
	0%	3.30%	13.30%	23.30%	33.30%	43.30%	53.30%	63.30%	73.30%	83.30%	93.30%	100%	
Dataset 1	100%	100%	100%	100%	100%	100%	100%	100%	78%	100%	100%	98%	98.97%
Dataset 2	100%	100%	100%	66%	100%	100%	100%	100%	100%	100%	100%	100%	98.76%

**Table 4:** Online fault detection accuracies of soft faults in the cable.

Dataset	Degrees of Damage												Overall
	Normal	Conductor (1- α in Percentage)										Shield	
	0%	3.30%	13.30%	23.30%	33.30%	43.30%	53.30%	63.30%	73.30%	83.30%	93.30%	100%	
Dataset 1	97%	100%	100%	100%	100%	100%	100%	100%	100%	100%	95%	100%	98.00%
Dataset 2	95%	85%	100%	85%	100%	100%	40%	10%	75%	100%	95%	100%	89.00%

**Table 5:** Comparison with other fault diagnosis methods.

	Proposed Method	Mechanical (EAB)	Chemical (OIT)	Electrical (Tan δ, PD)	TDR	FDR	TFDR	SSTDR
Soft fault detection capability	O	Δ	Δ	Δ	Δ	Δ	O	Δ
Applicable to very short cable	O	O	O	O	X	X	X	X
Online diagnosis	O	X	X	Δ	X	X	X	O
Offline diagnosis	O	O	O	O	O	O	O	O
No prior domain knowledge required	O	X	X	X	X	X	X	X
Nonintrusive and nondestructive	O	X	X	O	O	O	O	O
Applicable to installed cables	O	X	X	O	O	O	O	O

## Data Availability

The data presented in this study are available on request from the corresponding author. The data are not publicly available because it is company confidential information.
